# Combined Donor-Recipient Obesity and the Risk of Graft Loss After Kidney Transplantation

**DOI:** 10.3389/ti.2022.10656

**Published:** 2022-09-29

**Authors:** Faisal Jarrar, Karthik K. Tennankore, Amanda J. Vinson

**Affiliations:** ^1^ Faculty of Medicine, Dalhousie University, Halifax, NS, Canada; ^2^ Division of Nephrology, Department of Medicine, Nova Scotia Health Authority, Halifax, NS, Canada

**Keywords:** graft loss, weight mismatch, obesity, kidney transplant outcomes, body mass index, obesity pairing

## Abstract

**Background:** As the prevalence of obesity increases globally, appreciating the effect of donor and recipient (DR) obesity on graft outcomes is of increasing importance.

**Methods:** In a cohort of adult, kidney transplant recipients (2000–2017) identified using the SRTR, we used Cox proportional hazards models to examine the association between DR obesity pairing (body mass index (BMI) >30 kg/m^2^), and death-censored graft loss (DCGL) or all-cause graft loss, and logistic regression to examine risk of delayed graft function (DGF) and ≤30 days graft loss. We also explored the association of DR weight mismatch (>30 kg, 10-30 kg (D>R; D<R) and <10 kg (D = R)) with each outcome, stratifying by DR obesity pairing.

**Results:** Relative to non-obese DR, obese DR were highest risk for all outcomes (DCGL: HR 1.26, 95% CI 1.22–1.32; all-cause graft loss: HR 1.09, 95% CI 1.06–1.12; DGF: OR 1.98, 95% CI 1.89–2.08; early graft loss: OR 1.34, 95% CI 1.19–1.51). Donor obesity modified the risk of recipient obesity and DCGL [*p* = 0.001] and all-cause graft loss [*p* < 0.001] but not DGF or early graft loss. The known association of DR weight mismatch with DCGL was attenuated when either the donor or recipient was obese.

**Conclusion:** DR obesity status impacts early and late post-transplant outcomes.

## GRAPHICAL ABSTRACT

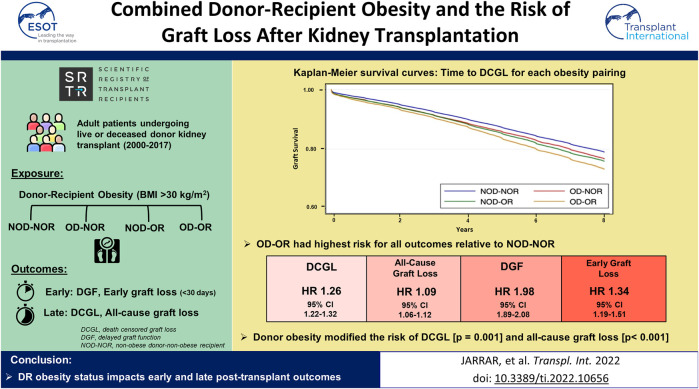

## Introduction

Obesity has become a major public health concern worldwide (1), with data classifying more than one third of adults as obese in the United States (2). The global rise in obesity is reflected in the kidney transplant population, with the proportion of recipients with a body mass index (BMI) in excess of 30 kg/m^2^ doubling every 15 years (3). As obesity rates increase in the general population, the number of obese transplant candidates and kidney donors, both living and deceased, is also expected to increase (4).

The increased prevalence of obesity has important implications for both kidney transplant recipients and transplant programmes. Although not considered a contraindication for kidney transplantation according to most clinical practice guidelines (5), recipient obesity is associated with increased risk of death-censored graft loss (DCGL) (6, 7, 8, 9, 10), delayed graft function (DGF) (6, 11, 12, 13, 14), increased peri- and post-operative complications (6, 15, 16) and prolonged hospitalizations (7, 8). Meanwhile, donor obesity has been linked with increased incidence of recipient DGF and DCGL (11, 17, 18), though its exact influence on graft outcomes is less clear. No studies to date have assessed the potential interaction between donor and recipient obesity on graft outcomes. Importantly, weight mismatch between kidney donors and recipients (DR) has been shown to associate with graft outcomes; recipients receiving organs from relatively smaller donors experience significantly worse outcomes than those receiving kidneys from weight-matched or larger donors (19, 20, 21, 22, 23). However, whether donor and/or recipient obesity modifies the association between DR weight mismatch and transplant outcomes has not been previously examined.

In this study, we aimed to describe the changing prevalence of donor and recipient obesity at the time of transplantation and explore whether *combined* DR obesity status impacts early (DGF, ≤30 day graft loss) and/or late (DCGL, all-cause graft loss) post-transplant outcomes. We also explored whether DR obesity status modifies the known relationship between DR weight mismatch and graft outcomes after kidney transplantation.

## Methods

### Subject Selection

We conducted a retrospective cohort study of adult patients who received a first living or deceased donor kidney transplant in the United States (US) between 1 January 2000, and 31 December 2016, identified using the Scientific Registry of Transplant Recipients (SRTR) database. Exclusion criteria included those <18 years of age, those receiving a second transplant, or those missing either donor or recipient data for weight or body mass index (BMI). Donors and recipients with BMI values <10 and >100 kg/m^2^ were excluded, as these were assumed to represent coding errors.

### Exposure

The primary exposure was donor and/or recipient obesity status. Obesity status was dichotomized at a BMI cut point of >30 kg/m^2^ versus ≤30 kg/m^2^ according to standard guidelines (24) to identify four DR obesity pairings: i. non-obese DR (NOD-NOR), ii. obese donor-non obese recipient (OD-NOR), iii. non obese donor-obese recipient (NOD-OR), and iv. obese DR (OD-OR).

A secondary exposure was combined donor and/or recipient obesity and DR weight mismatch. We categorized DR absolute weight difference as >30 kg, 10-30 kg (donor < recipient, D<R; or donor > recipient, D>R) and <10 kg (D = R) as per previous literature (19), stratified by the four aforementioned DR obesity pairings (NOD-NOR, OD-NOR, NOD-OR and OD-OR).

### Outcome

The primary outcome was death-censored graft loss (DCGL). Graft loss was defined as need for return to chronic dialysis or repeat transplantation. Secondary outcomes included the composite of graft failure or death (i.e., all-cause graft loss), delayed graft function (DGF), defined as need for dialysis within the first 7 days following transplantation, and early (≤30 days) graft loss. Censoring occurred at losses to follow-up and at the date of last follow-up.

### Data Collection

We adjusted for known literature predictors of graft loss including donor and recipient age, race, and sex, recipient end-stage kidney disease (ESKD) cause, dialysis vintage, pre-emptive status, cold-ischemia time (CIT), previous kidney transplant, human leukocyte antigen (HLA) mismatch (MM), peak panel reactive antibody (PRA), and recipient medical comorbidities including type 2 diabetes, hypertension, coronary artery disease and peripheral vascular disease. These co-variates were selected *a priori.* For the primary analysis, missing data was treated by case wise deletion.

The SRTR data system includes data on all donor, wait-listed candidates, and transplant recipients in the US, submitted by the members of the Organ Procurement and Transplantation Network (OPTN). The Health Resources and Services Administration (HRSA), US Department of Health and Human Services provides oversight to the activities of the OPTN and SRTR contractors.

### Analysis

Descriptive statistics were reported for baseline characteristics. Means and standard deviations and medians and interquartile range were used for continuous normal and continuous non-normally distributed variables. Baseline donor and recipient characteristics were reported for all patients in each of the DR obesity pairing groups.

#### Primary Analysis

##### Temporal Changes in DR Obesity Pairing Over Time

We examined temporal trends in the incidence of each DR obesity pairing at the time of transplantation over the study period.

##### Association of DR Obesity Pairing With DCGL

For the outcome of DCGL, we used a multivariable Cox proportional hazards model to determine the adjusted hazard ratio (HR) for DCGL for each DR obesity pairing (OD-NOR; NOD-OR; OD-OR), relative to NOD-NOR. Time to DCGL was demonstrated visually using Kaplan Meier survival curves. Proportionality was confirmed with visual examination of log-log plots.

#### Secondary Analyses

##### Association of DR Obesity Pairing With Secondary Outcomes

In a secondary analysis, we used a multivariable Cox proportional hazards model to determine the adjusted HR for all-cause graft failure for each DR obesity pairing (OD-NOR; NOD-OR; OD-OR), relative to NOD-NOR. Multivariable logistic regression was used to determine the adjusted odds ratio (OR) for the outcomes of DGF and early (≤30 days) graft loss associated with each DR obesity pairing relative to NOD-NOR. Finally, we determined if donor obesity modified the association of recipient obesity with each of DCGL, all-cause graft loss, DGF and early graft loss, by including an interaction term between donor and recipient obesity status in each regression model.

##### Association of Combined DR Weight Mismatch & Obesity Status With DCGL

For the outcome of DCGL, we used multivariable Cox proportional hazards models to determine the adjusted relative hazard ratio (HR) for each DR weight mismatch category relative to weight-matched DR (<10 kg absolute weight difference), stratified by DR obesity status. Weight-matched NOD-NOR was the reference category for all comparisons, irrespective of DR obesity status. Proportionality was confirmed with visual examination of log-log plots.

##### Association of Combined DR Weight Mismatch & Obesity Status With Secondary Outcomes

We repeated the above analysis examining DR weight mismatch stratified by DR obesity status to examine the outcome of all-cause graft loss. We also examined the effect of combined DR obesity and weight mismatch on DGF and early graft loss, using multivariable logistic regression, adjusting for the same factors listed above.

#### Sensitivity Analyses and Subgroup Analysis

We repeated our primary analysis (DR obesity pairing) for the following:

(i) Adjusting for era effect for the outcome of DCGL.
(ii) Excluding donors and recipients with a BMI <18 for early and late graft outcomes.
(iii) Adjusting for donation after circulatory death (DCD) vs**.** donation after neurologic death (DND) status for the outcome of DCGL in deceased donor transplant recipients.
(iv) Adjusting for donor kidney side (right vs. left) for early and late outcomes.

We repeated our secondary analysis (combined DR weight mismatch & obesity status) for:

(i) Combined DR weight mismatch and obesity status using a reference category of weight-matched DR (D = R) within each DR obesity pairing (as opposed to D = R NOD-NOR).
(ii) Combined DR weight mismatch and obesity status separately in living donors and deceased donors.
(iii) Using DR height mismatch instead of weight mismatch. For this analysis, we categorized DR absolute height difference as >15 cm, 5-15 cm (D<R; D>R) and <5 cm (D = R), as per previous literature (20). A <5 cm difference between donor and recipient height was used as the reference category for height mismatch. Similar to the primary analysis, we examined the association of DR height mismatch with DCGL within each DR obesity pairing.
(iv) Using higher BMI cut points (>35 kg/m^2^ and >40 kg/m^2^) to define DR obesity status; the reference category was patients with a BMI of 18–25 kg/m^2^.

Ethics approval for this study was provided through the Nova Scotia Health Research Ethics Board. All statistical analyses were performed using Stata version 13.1 (Stata Corp., College Station, TX). For statistical comparisons, a *p* < 0.05 was deemed the threshold for statistical significance.

## Results

### Baseline Characteristics

**FIGURE 1:**
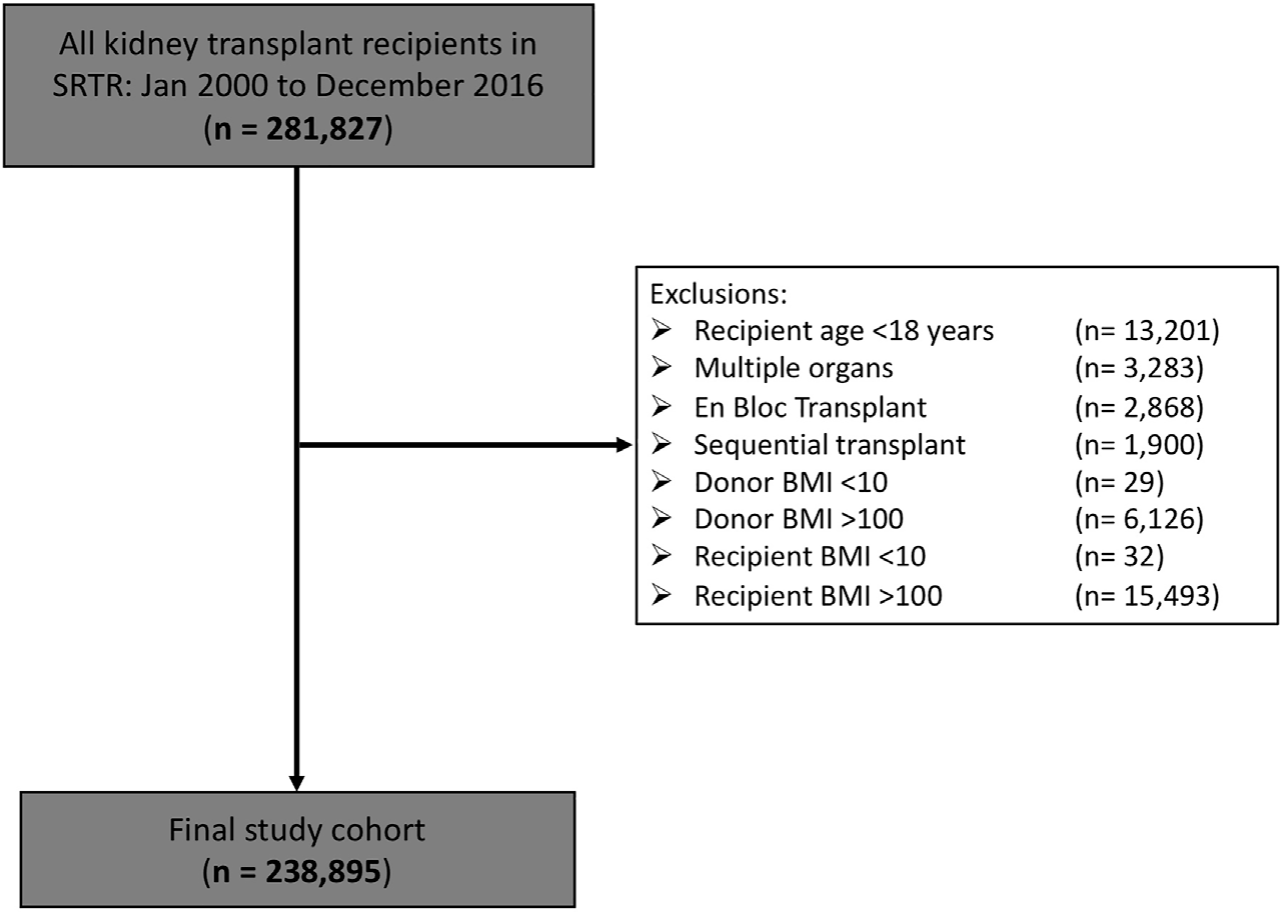
Final study cohort following exclusions.

Our final study cohort consisted of 238,895 kidney transplant recipients (Figure 1). Baseline characteristics are shown in Table 1. A total of 154,125 (64.5%) were from deceased donors and 84,770 (35.5%) from living donors. Mean donor and recipient BMIs were 27.1 ± 6.0 kg/m^2^ and 27.7 ± 5.6 kg/m^2^, respectively, with 40.0% and 49.7% of donors and recipients noted to be obese, respectively. Median absolute DR weight difference was −2.10 kg (Q1-Q3 −19.26 to 14.80 kg); recipients being slightly larger than donors. Overall, DCGL occurred in 30,132 patients (12.6%), all-cause graft loss in 82,372 (34.9%), DGF in 83,374 (18.1%) and early graft loss in 4778 (2%). Median follow-up time was 4.15 years (Q1–Q3 1.97–7.71 years).

**TABLE 1:** Baseline characteristics by donor-recipient obesity pairing.

Characteristics	Categories			
N = 238,895 (%)	NOD-NOR	OD-NOR	NOD-OR	OD-OR
	N = 123,449 (51.7)	N = 38,969 (16.3)	N = 53,964 (22.6)	N = 22,513 (9.4)
Donor age (Q1, Q3)	39 (26, 50)	43 (33, 52)	40 (27, 51)	43 (33, 52)
Recipient age (Q1, Q3)	51 (39, 60)	53 (41, 62)	53 (43, 61)	54 (44, 61)
Donor sex (F)	57,937 (46.9)	19,357 (49.7)	24,145 (44.7)	11,360 (50.5)
Recipient sex (F)	47,994 (38.9)	14,876 (38.2)	21,739 (40.3)	8,994 (40.0)
Donor race				
White	102,958 (83.4)	32,371 (83.1)	45,214 (83.8)	18,481 (82.1)
Black	14,613 (11.8)	5,585 (14.3)	7,016 (13.0)	3,497 (15.5)
Other	5,863 (4.8)	1,008 (2.6)	1,727 (3.2)	534 (2.4)
Recipient race				
White	85,145 (69.0)	25,557 (65.6)	36,067 (66.8)	14,695 (65.3)
Black	27,699 (22.44)	10,315 (26.5)	15,628 (29.0)	6,904 (30.7)
Other	10,605 (8.6)	3,097 (8.0)	2,266 (4.2)	914 (4.06)
Pre-emptive	24,115 (19.5)	6,373 (16.4)	9,423 (17.5)	3,795 (16.9)
HLA MM				
0	11,179 (9.1)	3,161 (8.1)	4,436 (8.2)	1,679 (7.5)
1	4,678 (3.8)	1,255 (3.2)	1,711 (3.2)	741 (3.3)
2	11,279 (9.1)	3,179 (8.2)	4,166 (7.7)	1,893 (8.4)
3	22,529 (18.3)	6,790 (17.4)	9,271 (17.2)	4,126 (18.3)
4	26,176 (21.2)	8,810 (22.6)	12,282 (22.8)	5,045 (22.4)
5	30,677 (24.9)	10,401 (26.7)	14,289 (26.5)	5,962 (26.5)
6	15,979 (12.9)	5,165 (13.3)	7,411 (13.7)	2,955 (13.1)
Previous transplant	17,333 (14.0)	5,389 (13.8)	4,670 (8.7)	1,768 (7.9)
Recipient diabetes	31,117 (25.2)	11,157 (28.6)	23,003 (42.6)	9,983 (44.3)
Recipient hypertension	93,868 (76.0)	29,970 (76.9)	41,896 (77.6)	17,436 (77.5)
Cause of ESRD				
Diabetes	24,229 (19.6)	8,788 (22.6)	17,906 (33.2)	7,822 (34.7)
Glomerulonephritis	32,830 (26.6)	9,391 (24.1)	11,637 (21.6)	4,628 (20.6)
PCKD	12,610 (10.2)	3,754 (9.6)	4,617 (8.6)	1,807 (8.0)
HTN	28,089 (22.8)	9,670 (24.8)	12,607 (23.4)	5,462 (24.3)
Hereditary	2,943 (2.4)	820 (2.1)	671 (1.2)	257 (1.1)
Drugs	2,897 (2.4)	841 (2.2)	824 (1.5)	350 (1.6)
Other	14,295 (11.6)	4,074 (10.5)	4,198 (7.8)	1,611 (7.2)
Median CIT (Q1, Q3)	11.5 (2.0,19.4)	13.2 (4.0, 20.7)	12.45 (2.75, 20.0)	12.48 (2.71, 20.0)
DR weight mismatch				
D>R, 10–30 kg (N = 48,908)	28,657 (23.3)	14,031 (36.0)	1,216 (2.3)	5,004 (22.2)
D>R, >30 kg (N = 25,552)	6,293 (5.1)	16,936 (43.5)	55 (0.1)	2,268 (10.1)
D = R, <10 kg (N = 74,555)	49,896 (40.4)	6,991 (17.9)	9,478 (17.6)	8,190 (36.4)
D<R, 10–30 kg (N = 56,617)	29,908 (24.2)	958 (2.5)	20,433 (37.9)	5,318 (23.6)
D<R, >30 kg (N = 33,263)	8,695 (7.0)	53 (0.1)	22,782 (42.2)	1,711 (7.7)

Proportion missing: human leukocyte antigen mismatch (0.8%); pre-emptive (0.48%); recipient diabetes (0.87%); recipient hypertension (12.7%); end-stage renal disease (3.9%); PRA (18.0%); donor race (0.01%); recipient race (0.003%); donor BMI (1.7%); recipient BMI (2.9%); CIT (11.0%).

BMI, body mass index; ESRD, end-stage renal disease; HLA, human leukocyte antigen; HTN, hypertension; PCKD, polycystic kidney disease; CIT, cold ischemia time; NOD-NOR, non-obese donor-non-obese recipient; OD-NOR, obese-donor-non-obese recipient; NOD-OR, non-obese donor-obese recipient; OD-OR, obese-donor-obese-recipient.

### Temporal Changes in DR Obesity Pairing

**FIGURE 2:**
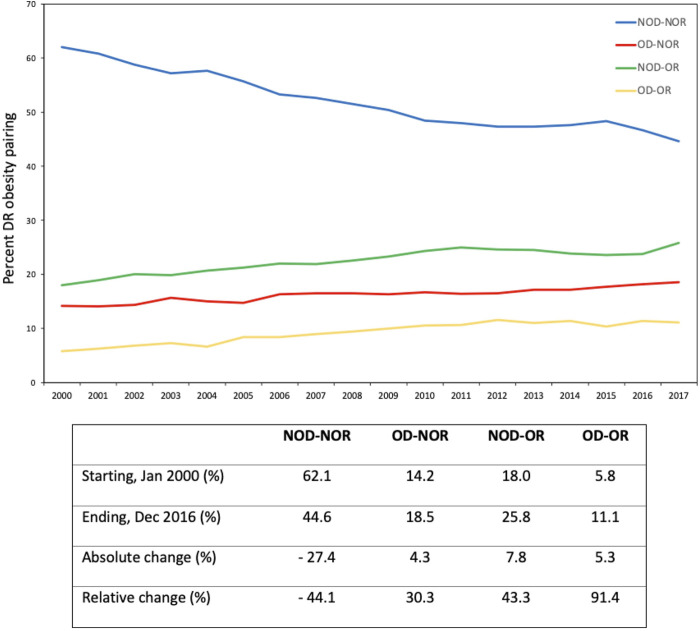
Temporal changes in donor-recipient obesity pairing over time. The accompanying table displays descriptive statistics for each of the donor-recipient obesity pairings.

There was a decrease in the incidence of NOD-NOR from 62% to 45% over time (Figure 2). Of the DR obesity pairings, NOD-OR had the greatest absolute increase over time (18% to 26%; 43.3% relative increase). OD-OR experienced the greatest relative increase over time, from 6% to 11% (91.4% relative increase).

### DR Obesity Pairing

#### DR Obesity and DCGL

**FIGURE 3:**
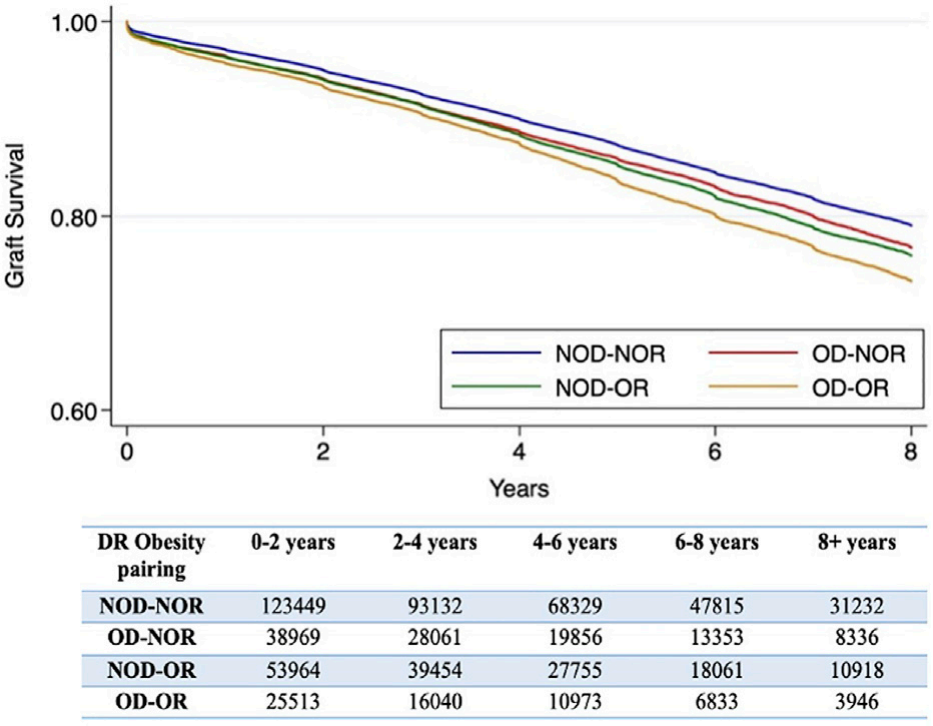
Kaplan-Meier survival curves for time to death-censored graft loss for each donor-recipient obesity pairing. A number at risk table is included below the figure. *The log-rank *p*-value is <0.001.

Examining the effect of DR obesity status on DCGL (relative to NOD-NOR), the adjusted relative hazard was highest in the OD-OR pairing (HR 1.24, 95% CI 1.19–1.30), Table 2. This was followed by NOD-OR (HR 1.16, 95% CI 1.12–1.20). OD-NOR pairing was not associated with risk of DCGL. The fully adjusted multivariable model is available in Supplementary Table S1A. Time to DCGL for each of the DR obesity pairings is shown in Figure 3.

**TABLE 2:** Adjusted risk for post-transplant adverse outcomes for each DR obesity pairing.

	DCGL	All-cause graft loss	DGF	Early (≤30 days) graft loss
	Hazard ratio (95% CI)	Hazard ratio (95% CI)	Odds ratio (95% CI)	Odds ratio (95% CI)
NOD-NOR	Ref.	Ref.	Ref.	Ref.
OD-NOR	1.01 (0.98–1.05)	0.99 (0.97–1.02)	**1.36 (1.31–1.42)**	**1.20 (1.08–1.34)**
NOD-OR	**1.16 (1.12–1.20)**	**1.05 (1.02–1.07)**	**1.49 (1.43–1.54)**	**1.19 (1.08–1.31)**
OD-OR	**1.24 (1.19–1.30)**	**1.08 (1.04–1.11)**	**1.98 (1.88–2.08)**	**1.32 (1.16–1.51)**

Green (HR < 1.0), yellow (HR 1-1.2), orange (HR 1.2-1.4), red (HR > 1.4) (Colors only apply to significant results).

Models were adjusted for known literature predictors of graft loss, including donor and recipient age, race, sex, recipient end-stage kidney disease (ESKD) cause, cold ischemia time (CIT), dialysis vintage, pre-emptive status, previous kidney transplant, human leukocyte antigen (HLA) mismatch, peak panel reactive antibody (PRA), and recipient medical comorbidities (coronary artery disease, hypertension, peripheral vascular disease, type 2 diabetes)*.*

NOD-NOR, non-obese donor-non-obese recipient; OD-NOR, obese-donor-non-obese recipient; NOD-OR, non-obese donor-obese recipient; OD-OR, obese-donor-obese-recipient; DCGL, death-censored graft loss; DGF, delayed graft function.

#### DR Obesity and Secondary Outcomes

Combined donor and recipient obesity (OD-OR) was also associated with the highest risk for all-cause graft loss, DGF and early graft loss, Table 2. OD-NOR pairing was associated with DGF and early graft loss but not with all-cause graft loss. NOD-OR pairing was associated with both early and late outcomes. The fully adjusted multivariable models are available in Supplementary Tables S1B–D.

Donor obesity modified the risk of recipient obesity on both DCGL (*p* = 0.001) and all-cause graft loss (*p* < 0.001), while no interaction was observed between donor and recipient obesity for DGF (*p* = 0.559) or early graft loss (*p* = 0.208).

### Combined DR Weight Mismatch & Obesity Pairing

#### Association With DCGL

Amongst NOD-NOR, both D>R by 10–30 kg (HR 0.94, 95% CI 0.90–0.99) and 30 kg (HR 0.84, 95% CI 0.77–0.92) were protective against DCGL and D<R by 10–30 kg (HR 1.12, 95% CI 1.07–1.17) and 30 kg (HR 1.42, 95% CI 1.33–1.52) were risk factors for DCGL versus no weight difference, Table 3. In all DR obesity pairings, there was a trend towards increased risk of DCGL as the recipient size increased relative to the donor and when either the donor or recipient were obese, D>R was no longer protective. In OD-OR, all DR weight mismatch categories were associated with an increased risk of DCGL relative to weight-matched NOD-NOR.

**TABLE 3:** Hazard ratios for death-censored graft loss for each DR weight mismatch category stratified by DR obesity status. Reference category used for all DR obesity pairings was weight-matched (D = R) NOD-NOR.

Hazard ratio for DCGL (95% CI)				
DR Weight Mismatch (kg)	NOD-NOR	OD-NOR	NOD-OR	OD-OR
	N = 123,449	N = 38,969	N = 53,964	N = 22,513
>30 (D>R)	**0.84 (0.77–0.92)**	0.99 (0.94–1.05)	0.54 (0.20–1.44)	**1.29 (1.15–1.46)**
10-30 (D>R)	**0.94 (0.90–0.99)**	1.05 (0.99–1.11)	1.14 (0.96–1.35)	**1.19 (1.09–1.30)**
< 10 (D = R)	Ref.	**1.15 (1.07–1.24)**	1.06 (0.99–1.14)	**1.24 (1.16–1.33)**
10-30 (D<R)	**1.12 (1.07–1.17)**	1.19 (0.98–1.46)	**1.12 (1.07–1.18)**	**1.38 (1.27–1.50)**
>30 (D<R)	**1.42 (1.33–1.52)**	1.88 (0.98–3.61)	**1.32 (1.26–1.39)**	**1.46 (1.28–1.67)**

Green (HR < 1.0), yellow (HR 1-1.2), orange (HR 1.2-1.4), red (HR > 1.4) (Colors only apply to significant results).

NOD-NOR, non-obese donor-non-obese recipient; OD-NOR, obese-donor-non-obese recipient; NOD-OR, non-obese donor-obese recipient; OD-OR, obese-donor-obese-recipient; DCGL, death-censored graft loss.

Models were adjusted for known literature predictors of graft loss, including donor and recipient age, race, sex, recipient end-stage kidney disease (ESKD) cause, cold ischemia time (CIT), dialysis vintage, pre-emptive status, previous kidney transplant, human leukocyte antigen (HLA) mismatch, peak panel reactive antibody (PRA), and recipient medical comorbidities (coronary artery disease, hypertension, peripheral vascular disease, type 2 diabetes)*.*

#### Association With Secondary Outcomes

Amongst NOD-NOR, D>R was not protective against all-cause graft loss, but a larger recipient than donor was significantly higher risk than no weight difference, Supplementary Table S2. Amongst OD-OR, all DR weight mismatch categories (except D>R by >30 kg) were higher risk for all-cause graft loss than a weight matched NOD-NOR; no significant association was seen for OD-NOR and NOD-OR.

Amongst NOD-NOR, a 30 kg difference between donor and recipient (D<R) was the highest risk for DGF (OR 1.24, 95% CI 1.14–1.34) relative to no weight mismatch, Supplementary Table S3. Though not always significant, when stratified by DR obesity status, all DR weight mismatch categories were associated with DGF. Risk of DGF was most pronounced for OD-OR and highest at extremes of weight mismatch (>30 kg difference) for both D>R and D<R.

Results for early graft loss are shown in Supplementary Table S4. D<R by 30 kg was highest risk in each DR obesity pairing.

### Sensitivity Analyses

#### Transplant Era Effect

When we repeated the primary analysis adjusting for transplant era, we found that the effects of DR obesity persisted and were similar to those seen in our primary analysis. The adjusted relative hazard was highest in the OD-OR pairing (HR 1.28, 95% CI 1.23–1.34), followed by NOD-OR (HR 1.18, 95% CI 1.14–1.21). OD-NOR pairing was not associated with risk of DCGL.

#### Exclusion of Donors and Recipients With BMI <18

When we repeated the primary analysis excluding donors and recipients with BMI <18, the same trends were observed for both early and late outcomes (Supplementary Table S5).

#### DND vs. DCD Status (Deceased Donors)

When we repeated our primary analysis adjusting for DCD vs**.** DND status in deceased donor transplant recipients, we found no significant association between DCD status and risk of DCGL (HR 0.97, 95% CI 0.92–1.02).

#### Donor Kidney Side

When we repeated our primary analysis adjusting for transplant kidney side, we found no significant association between right-sided donor transplants and risk of DCGL (HR 0.99, 95% CI 0.97–1.02) or all-cause graft loss (HR 0.99, 95% CI 0.97–1.01). A significant association was found between right-sided donor transplants and both DGF (OR 1.08, 95% CI 1.05–1.11) and early graft loss (OR 1.12, 95% CI 1.03–1.22).

#### Association of Combined DR Weight Mismatch & Obesity With DCGL; Modified DR Reference Category

**FIGURE 4:**
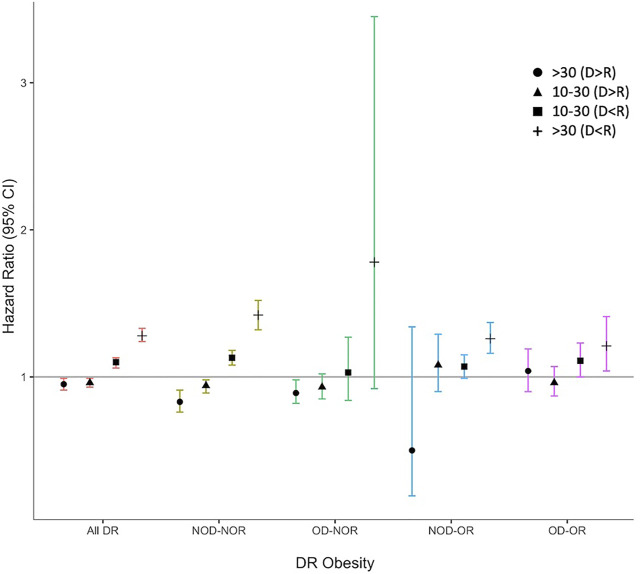
Hazard ratio plot for death-censored graft loss for combined donor-recipient weight mismatch, stratified by donor-recipient obesity. Models were adjusted for known literature predictors of graft loss, including donor and recipient age, race, sex, recipient end-stage kidney disease (ESKD) cause, cold ischemia time (CIT), dialysis vintage, pre-emptive status, previous kidney transplant, human leukocyte antigen (HLA) mismatch, peak panel reactive antibody (PRA), and recipient medical comorbidities (coronary artery disease, hypertension, peripheral vascular disease, type 2 diabetes).

When we used a weight matched reference category within each DR obesity pairing (as opposed to D = R in NOD-NOR for all comparisons), overall D>R was protective against DCGL and D<R was a risk for DCGL, Figure 4, Supplementary Table S6. In NOD-NOR, point estimates were more pronounced for D>R by 30 kg (HR 0.83, 95% CI 0.76–0.91) and D<R by 30 kg (HR 1.42, 95% CI 1.32–1.52) compared to D = R. Amongst OD-OR, DR weight mismatch was not associated with DCGL.

#### Height Mismatch

Amongst the entire cohort, risk of DCGL increased as recipient height increased relative to donor, though not all results reached statistical significance (Supplementary Table S7). A donor >15 cm taller than their recipient was protective against DCGL in the overall cohort (HR 0.91, 95% CI 0.87–0.94) and NOD-NOR (HR 0.89, 95% CI 0.84–0.95); this protective effect was not significant in any of the other DR obesity pairings.

#### Living vs. Deceased Donors

There was a trend towards increased risk of DCGL as the recipient-to-donor weight increased in most DR obesity pairings, though results did not always reach statistical significance (Supplementary Tables S8, S9). This analysis was limited by small subgroup sample sizes, particularly for OD-NOR in living donor transplants.

#### Extremes of BMI

Relative to NOD-NOR, risk of DCGL was highest for OD-OR using both >35 kg/m^2^ (HR 1.45, 95% CI 1.31–1.60) and >40 kg/m^2^ (HR 1.41, 95% CI 1.05–1.90) cut-offs, followed by NOD-OR (BMI ≥35 kg/m^2^: HR 1.26, 95% CI 1.18–1.35; BMI 40 kg/m^2^: HR 1.36, 95% CI 1.20–1.54). OD-NOR was not significantly associated with DCGL for either BMI cut-offs, (data not shown). Sample sizes were small in the OD-OR subgroup at BMI 40 kg/m^2^ (*n* = 203).

## Discussion

In this study, we describe the changing demographics of obesity at the time of kidney transplantation and explore how DR obesity pairing impacts early and late graft outcomes. We also investigate whether obesity status modifies the known relationship between DR weight mismatch and graft outcomes after kidney transplantation.

Previous studies have found a significant increase in the prevalence of overweight and obese recipients at time of transplantation (3, 16). We demonstrate a substantial increase in the prevalence of obesity in both kidney donors and recipients over time, with relative increases in NOD-OR transplants by 43.3% and OD-OR by over 91.4% over our study period.

When examining the effect of DR obesity pairing on late graft outcomes, OD-OR and NOD-OR were both associated with risk of DCGL and all-cause graft loss; OD-OR was highest risk for both outcomes. Isolated recipient obesity has been linked to a multitude of adverse graft outcomes, including DCGL (6, 25, 26) and early events including wound-related morbidity and acute rejection (27, 28), which likely compound the risk of long-term failure. Obesity is associated with chronic medical conditions including type 2 diabetes, cardiovascular disease, and chronic respiratory disorders, which are associated with increased morbidity and mortality in the general population and kidney transplant recipients (29, 30, 31, 32). Obesity also causes various structural, hemodynamic, and metabolic alterations in the kidney (33). It has been hypothesized that a kidney that is small for the metabolic needs of an individual may experience a triad of glomerular hypertension, hypertrophy, and hyperfiltration that eventually leads to progressive glomerulosclerosis, proteinuria, and loss of function (17, 33, 34, 35); these renal complications are seen in obesity-related glomerulopathy (ORG) (36, 37). Damage to transplanted kidneys may be caused by similar pathophysiologic mechanisms to those which occur in the native kidneys of obese patients, contributing to downstream adverse effects in recipients (38, 39). We demonstrate for the first time that donor obesity modifies the known association between recipient obesity and DCGL and all-cause graft loss. This interaction likely relates to additive harms when an obese donor kidney (with some element of pre-existing pre-terminal hyperfiltration and ORG) is transplanted into an obese recipient wherein pre-existing vascular disease, longer operative times and surgical complications may compound risk (17, 27).

Notably, risk of DCGL was more exaggerated than that of all-cause graft loss in both NOD-OR and OD-OR. This finding is in keeping with other studies which have shown a comparable mortality risk between obese recipients and those with a normal BMI (4, 6, 12). While this appears counter-intuitive given the greater burden of co-morbidities in obese individuals and the association of obesity with mortality in the general population (40), there are a number of possible explanations. First, the J-shaped relationship between BMI and survival in the prevalent dialysis population is important to consider, wherein both high and low BMIs are associated with increased mortality (41, 42). This likely reflects a combination of underlying comorbidity, protein-energy malnutrition, or the existence of a chronic inflammatory state as opposed to a directly protective effect of adiposity (41, 43). Second, renal transplant recipients have a substantial increased risk of cardiovascular morbidity by virtue of an accumulation of traditional and transplant-related risk factors (44, 45). It is thus possible that the additional mortality risk conferred by obesity is overshadowed by the significant cardiovascular risk in this unique population.

We found an increased risk of DGF when either the donor or recipient was obese, with the risk greatest in OD-OR. This is in agreement with previous retrospective studies which have separately correlated recipient and donor BMI with incidence of DGF (11, 12, 13, 14). DGF is a consequence of mostly, but not exclusively, nonimmunological factors (e.g., hypoxia during cold or warm ischemic periods) and ischemia-reperfusion–mediated immunological factors (46, 47). Previous studies have shown that obese recipients are more likely to experience protracted operative times, early post-operative complications (27, 28, 42), acute rejection (14) and prolonged warm ischemia times (48, 49). Donor obesity has also been linked with increased nephrectomy operation times as well as prolonged cold and warm ischemia times (18, 50). The association between BMI and ischemia-reperfusion injury has not been well studied, however, obesity is considered a proinflammatory environment marked by an increased activation of innate and adaptive immune responses (4). Adipocytes and immune cells within adipose tissue are known to produce proinflammatory cytokines including IL6, TNF-alpha and IL1-beta, while anti-inflammatory mediators are simultaneously suppressed (4, 51, 52). After transplant surgery, obesity-related proinflammatory cytokines may stimulate an exaggerated ischemia-reperfusion injury–mediated immunological response, contributing to both DGF and early graft loss. Further, venous thromboembolism, risk of which is higher in obese patients (53), may contribute to the early outcomes seen in obese recipients (54).

Our analysis demonstrates an attenuation of the protective effects of a larger donor than recipient (16, 17, 18, 19, 20, 24, 55) when either the donor or recipient is obese. This finding may similarly be explained by the nephron underdosing hypothesis (39, 56) whereby the relatively smaller renal mass in smaller donors results in increased single nephron glomerular filtration rate and increased risk of hyperfiltration injury over time (34, 38, 57, 58, 59). While recipients are typically protected by larger donors because of the greater nephron load afforded, there is likely paradoxical nephron underdosing when larger donors are obese. Nephron load is thought to be a correlate of lean body mass, not actual body mass in obese individuals, (33, 36) and as such, larger donors due to increased adiposity would not be expected to yield a greater nephron supply. Additionally, glomerular hyperfiltration, which occurs in the context of the increased metabolic needs of obesity, may lead to the development of glomerulomegaly and glomerulosclerosis in a manner analogous to that described in reduced renal mass states (36, 37, 60). This has been observed in patients with biopsy-proven ORG (37). Obesity therefore mitigates the protective association seen when donors are larger than their recipients given the combined effect of lower nephron density per unit mass and underlying glomerulosclerosis in the obese donor kidney at the time of donation.

Interpretation of the findings regarding obesity and graft outcomes requires caution. Although this study demonstrates the potential detriments of donor and recipient obesity on outcomes following transplantation, we do not suggest discard of obese donor kidneys or that obese recipients be declined access to transplantation. Evidence suggests that in most cases, kidney transplantation in obese patients affords better survival than remaining on dialysis (4). Glanton et al. reported doubled mortality rates for obese patients who stayed on the waiting list compared to those who received a kidney transplant, though this survival benefit was not achieved in patients with BMI ≥40 kg/m^2^ (61). Our study highlights the importance of counseling potential recipients on achieving a healthy pre-transplant BMI to optimize post-transplant outcomes.

While likely of benefit, there are insufficient data to assess the impact of pre-transplant interventions, such as planned weight reduction strategies, among potential recipients. The role of bariatric surgery in the dialysis population and transplant candidates is becoming an increasingly salient issue, with many studies showing promising results (62, 63, 64). Pending more evidence, encouraging kidney transplant candidates living with obesity to lose weight and have their nutritional status supervised by a multidisciplinary weight-management team remains important (5). Obese transplant candidates should continue to be carefully optimized prior to surgery to minimize peri- and post-operative morbidity and post-operative graft injury. This may include strategic pairing of donors and recipients to minimize additive insults from suboptimal DR weight mismatch and obesity pairing.

There are several limitations to our study for consideration. First, while BMI is often used as a surrogate marker of obesity and suitability for kidney transplantation, some studies have shown waist-to-hip ratio and waist circumference to be stronger predictors of cardiovascular death than BMI (65). Waist circumference is currently not collected in the SRTR, but its application and comparison to BMI in future analyses is important. Second, the internal consistency of BMI in donors and recipients may be questioned; it is plausible that an elevated BMI in donors and recipients is associated with significant differences in lean body masses. As demonstrated by previous literature, many patients with ESKD are in a catabolic state manifested by a combination of underlying comorbidity, protein-energy malnutrition, and a chronic inflammatory state (43). In such states, a higher BMI may reflect lower overall risk. As such, examination of potentially more reliable clinical markers, such as BSA, are warranted in future investigations. Third, our study dichotomized DR obesity at a BMI cut point of 30 kg/m^2^ as defined by earlier literature (66). Ideally, further sub-categorization of BMI would be undertaken to better understand how varying degrees of donor and/or recipient obesity influence graft outcomes, however, this as demonstrated by our sensitivity analysis examining OD-OR defined using a BMI cut point of 40 kg/m^2^ limited the available sample sizes and the validity of the results. Additionally, we could not access any histologic parameters of the allograft such as implantation biopsy, percentage of global glomerulosclerosis, or health of the tubulointerstitium, which could provide important insights on histopathologic changes related to obesity. A prospective study at an appropriate center could allow for exploration of implantation biopsies at the time of organ retrieval. Moreover, immunosuppressive data including details regarding changes over time, are not robustly captured by the SRTR and were therefore not included in our multivariable models. Finally, we could not access specific causes of graft loss; these may have provided pathophysiologic explanations as to how DR obesity status influences early and late graft loss. As such, we could not establish the relative impact of specific factors for a given recipient on graft loss. We also could not access donors’ cause of death as this is not reliably reported in the SRTR.

In summary, we report an increased proportion of obese donors and recipients between 2000 and 2017, with the greatest relative increase in OD-OR followed by NOD-OR. We demonstrate the combined exposure of an obese donor and obese recipient to be associated with the greatest risk of short and long-term complications after transplant. Finally, we demonstrate that donor and/or recipient obesity attenuates the protective signal typically seen in the setting of a larger donor-to-recipient size. Our findings highlight the importance of informed consent procedures for obese donors and transplant candidates. Further, our data indicate that obesity status should be considered when considering the implications of DR weight matching.

## Data Availability

Publicly available datasets were analyzed in this study. This data can be found here: Scientific Registry of Transplant Recipients.

## Abbreviations

SRTR, Scientific Registry of Transplant Recipients; BMI, Body Mass Index; DCGL, Death-censored graft loss; DGF, Delayed graft function; DR, Donor and recipient; HR, Hazard ratio; OR, Odds ratio; NOD-NOD, Non-obese donor and recipient; OD-NOR, Obese donor-non obese recipient; NOD-OR, Non obese donor-obese recipient; OD-OR, Obese donor and recipient; ESKD, End-stage kidney disease; HLA MM, Human leukocyte antigen mismatch; PRA, Peak panel reactive antibody.
