# Supplementary material for: Combined Donor-Recipient Obesity and the Risk of Graft Loss After Kidney Transplantation
Source: Transpl Int. 2022 Sep 29;35:10656. doi: 10.3389/ti.2022.10656 (PMC9556700; doi:10.3389/ti.2022.10656)
Supplement: Supplementary file 1 [file DataSheet1.docx]

**Supplemental Table of Contents**

Table 1a. Multivariable cox proportional hazards model for factors associated with DCGL

Table 1b. Multivariable cox proportional hazards model for factors associated with all-cause graft loss

Table 1c. Multivariable logistic regression model for factors associated with delayed graft function

Table 1d. Multivariable logistic regression model for factors associated with early graft loss

Table 2. Hazard ratios for all-cause graft loss for each DR weight mismatch category stratified by DR obesity status

Table 3. Odds ratios for DGF for each DR weight mismatch category stratified by DR obesity status

Table 4. Odds ratios for early graft loss for each DR weight mismatch category stratified by DR obesity status

Table 5. Hazard ratios for DCGL by DR obesity status excluding donors or recipients with BMI <18

Table 6. Hazard ratios for DCGL for each DR weight mismatch category, stratified by DR obesity status (reference category used was weight-matched (D=R) NOD-NOR).

Table 7. Hazard ratios for DCGL for each DR height mismatch, category stratified by DR obesity status

Table 8. Hazard ratios for DCGL for each DR weight mismatch category stratified by DR obesity status for deceased donors

Table 9. Hazard ratios for DCGL for each DR weight mismatch category stratified by DR obesity status for living donors

**Supplemental Table 1a:** Multivariable cox proportional hazards model for factors associated with DCGL

| Patient Characteristics | HR | 95% CI |
| --- | --- | --- |
| DR Obesity Pairing | | |
| NOD-NOR | Ref. |  |
| OD-NOR | 1.01 | 0.98-1.05 |
| NOD-OR | 1.16 | 1.12-1.20 |
| OD-OR | 1.24 | 1.19-1.30 |
| Donor Sex | | |
| Male | Ref. |  |
| Female | 1.10 | 1.08-1.13 |
| Recipient Sex | | |
| Male | Ref. |  |
| Female | 0.99 | 0.97-1.02 |
| Donor race (black) | 1.27 | 1.23-1.32 |
| Recipient race (black) | 1.41 | 1.37-1.45 |
| CIT | 1.01 | 1.01-1.01 |
| Recipient co-morbidities | | |
| CAD | 1.10 | 1.05-1.15 |
| PVD | 1.11 | 1.05-1.18 |
| HTN | 0.92 | 0.88-0.96 |
| Type 2 diabetes | 1.11 | 1.05-1.17 |
| PRA group |  |  |
| Group 1 | 1.05 | 1.01-1.10 |
| Group 2 | 1.15 | 1.09-1.22 |
| HLA Mismatch |  |  |
| 1 | 1.21 | 1.11-1.31 |
| 2 | 1.35 | 1.26-1.44 |
| 3 | 1.41 | 1.33-1.49 |
| 4 | 1.42 | 1.34-1.50 |
| 5 | 1.52 | 1.44-1.60 |
| 6 | 1.63 | 1.54-1.73 |
| Living donor | 0.74 | 0.71-0.77 |
| Cause of ESRD |  |  |
| Type 2 diabetes | 0.97 | 0.92-1.03 |
| PCKD | 0.69 | 0.66-0.73 |
| HTN | 1.07 | 1.03-1.11 |
| Other | 0.95 | 0.92-0.99 |
| Previous kidney transplant | 1.16 | 1.11-1.20 |
| Pre-emptive transplant |  |  |
| Yes | Ref. |  |
| No | 1.41 | 1.36-1.47 |

Abbreviations: ESRD, end-stage renal disease; HLA, human leukocyte antigen; HTN, hypertension; PCKD, polycystic kidney disease; CIT, cold ischemia time; PRA, panel reactive antibody. DR, donor-recipient; NOD-NOR, non-obese donor-non-obese recipient; OD-NOR, obese donor-non-obese recipient; NOD-OR, non-obese donor-obese recipient; OD-OR, obese donor-obese recipient

**Supplemental Table 1b:** Multivariable cox proportional hazards model for factors associated with all-cause graft loss

| Patient Characteristics | HR | 95% CI |
| --- | --- | --- |
| DR Obesity Pairing | | |
| NOD-NOR | Ref. |  |
| OD-NOR | 0.99 | 0.97-1.02 |
| NOD-OR | 1.05 | 1.02-1.07 |
| OD-OR | 1.08 | 1.04-1.11 |
| Donor Sex | | |
| Male | Ref. |  |
| Female | 1.06 | 1.04-1.08 |
| Recipient Sex | | |
| Male | Ref. |  |
| Female | 0.95 | 0.93-0.97 |
| Donor race (black) | 1.16 | 1.13-1.19 |
| Recipient race (black) | 1.15 | 1.13-1.79 |
| CIT | 1.01 | 1.00-1.01 |
| Recipient co-morbidities | | |
| CAD | 1.24 | 1.21-1.28 |
| PVD | 1.23 | 1.19-1.27 |
| HTN | 0.90 | 0.87-0.92 |
| Type 2 diabetes | 1.24 | 1.20-1.29 |
| PRA group |  |  |
| Group 1 | 1.01 | 0.98-1.04 |
| Group 2 | 1.08 | 1.04-1.13 |
| HLA Mismatch |  |  |
| 1 | 1.12 | 1.06-1.18 |
| 2 | 1.19 | 1.14-1.24 |
| 3 | 1.20 | 1.15-1.24 |
| 4 | 1.17 | 1.13-1.21 |
| 5 | 1.21 | 1.17-1.26 |
| 6 | 1.27 | 1.22-1.32 |
| Living donor | 0.75 | 0.73-0.77 |
| Cause of ESRD |  |  |
| Type 2 diabetes | 1.15 | 1.10-1.19 |
| PCKD | 0.70 | 0.67-0.73 |
| HTN | 1.13 | 1.11-1.16 |
| Other | 1.11 | 1.08-1.15 |
| Previous kidney transplant | 1.16 | 1.12-1.19 |
| Pre-emptive transplant |  |  |
| Yes | Ref. |  |
| No | 1.43 | 1.39-1.47 |

Abbreviations: ESRD, end-stage renal disease; HLA, human leukocyte antigen; HTN, hypertension; PCKD, polycystic kidney disease; CIT, cold ischemia time; PRA, panel reactive antibody. DR, donor-recipient; NOD-NOR, non-obese donor-non-obese recipient; OD-NOR, obese donor-non-obese recipient; NOD-OR, non-obese donor-obese recipient; OD-OR, obese donor-obese recipient

**Supplemental Table 1c:** Multivariable logistic regression model for factors associated with delayed graft function

| Patient Characteristics | OR | 95% CI |
| --- | --- | --- |
| DR Obesity Pairing | | |
| NOD-NOR Ref. | | |
| OD-NOR 1.36 1.31-1.42 | | |
| NOD-OR 1.49 1.43-1.54 | | |
| OD-OR 1.98 1.88-2.08 | | |
| Donor Sex | | |
| Male | Ref. |  |
| Female | 0.85 | 0.82-0.87 |
| Recipient Sex | | |
| Male | Ref. |  |
| Female | 0.73 | 0.71-0.75 |
| Donor race (black) | 0.92 | 0.88-0.98 |
| Recipient race (black) | 1.41 | 1.36-1.46 |
| CIT | 1.03 | 1.02-1.03 |
| Recipient co-morbidities | | |
| CAD | 1.12 | 1.06-1.17 |
| PVD | 1.10 | 1.04-1.17 |
| HTN | 0.90 | 0.86-0.95 |
| Type 2 diabetes | 1.18 | 1.11-1.25 |
| PRA group |  |  |
| Group 1 | 1.05 | 1.00-1.10 |
| Group 2 | 1.27 | 1.20-1.36 |
| HLA Mismatch |  |  |
| 1 | 1.05 | 0.94-1.16 |
| 2 | 1.24 | 1.14-1.35 |
| 3 | 1.35 | 1.20-1.37 |
| 4 | 1.29 | 1.22-1.37 |
| 5 | 1.36 | 1.28-1.44 |
| 6 | 1.46 | 1.37-1.56 |
| Living donor | 0.23 | 0.22-0.25 |
| Cause of ESRD |  |  |
| Type 2 diabetes | 1.12 | 1.04-1.20 |
| PCKD | 0.92 | 0.86-0.97 |
| HTN | 1.16 | 1.11-1.21 |
| Other | 1.06 | 1.01-1.12 |
| Previous kidney transplant | 1.20 | 1.14-1.26 |
| Pre-emptive transplant |  |  |
| Yes | Ref. |  |
| No | 3.85 | 3.59-4.13 |

Abbreviations: ESRD, end-stage renal disease; HLA, human leukocyte antigen; HTN, hypertension; PCKD, polycystic kidney disease; CIT, cold ischemia time; PRA, panel reactive antibody. DR, donor-recipient; NOD-NOR, non-obese donor-non-obese recipient; OD-NOR, obese donor-non-obese recipient; NOD-OR, non-obese donor-obese recipient; OD-OR, obese donor-obese recipient

**Supplemental Table 1d:** Multivariable logistic regression model for factors associated with early graft loss

| Patient Characteristics | OR | 95% CI |
| --- | --- | --- |
| DR Obesity Pairing | | |
| NOD-NOR | Ref. |  |
| OD-NOR | 1.20 | 1.08-1.34 |
| NOD-OR | 1.19 | 1.08-1.31 |
| OD-OR | 1.32 | 1.16-1.51 |
| Donor Sex | | |
| Male | Ref. |  |
| Female | 1.02 | 0.94-1.10 |
| Recipient Sex | | |
| Male | Ref. |  |
| Female | 1.10 | 1.01-1.19 |
| Donor race (black) | 1.05 | 0.93-1.18 |
| Recipient race (black) | 1.05 | 0.95-1.15 |
| CIT | 1.01 | 1.01-1.02 |
| Recipient co-morbidities | | |
| CAD | 1.09 | 0.95-1.25 |
| PVD | 1.29 | 1.10-1.51 |
| HTN | 0.85 | 0.76-0.96 |
| Type 2 diabetes | 0.94 | 0.80-1.10 |
| PRA group |  |  |
| Group 1 | 1.03 | 0.91-1.16 |
| Group 2 | 1.18 | 1.00-1.39 |
| HLA Mismatch |  |  |
| 1 | 1.21 | 0.94-1.56 |
| 2 | 1.26 | 1.03-1.54 |
| 3 | 1.29 | 1.09-1.53 |
| 4 | 1.28 | 1.09-1.50 |
| 5 | 1.27 | 1.08-1.49 |
| 6 | 1.39 | 1.17-1.65 |
| Living donor | 0.72 | 0.64-0.82 |
| Cause of ESRD |  |  |
| Type 2 diabetes | 1.09 | 0.91-1.31 |
| PCKD | 0.85 | 0.72-1.00 |
| HTN | 1.07 | 0.95-1.20 |
| Other | 1.26 | 1.11-1.42 |
| Previous kidney transplant | 1.35 | 1.19-1.52 |
| Pre-emptive transplant |  |  |
| Yes | Ref. |  |
| No | 1.04 | 0.93-1.17 |

Abbreviations: ESRD, end-stage renal disease; HLA, human leukocyte antigen; HTN, hypertension; PCKD, polycystic kidney disease; CIT, cold ischemia time; PRA, panel reactive antibody. DR, donor-recipient; NOD-NOR, non-obese donor-non-obese recipient; OD-NOR, obese donor-non-obese recipient; NOD-OR, non-obese donor-obese recipient; OD-OR, obese donor-obese recipient

**Supplemental Table 2:** Hazard ratios for all-cause graft loss for each DR weight mismatch category stratified by DR obesity status. Reference category used for all DR obesity pairings was weight-matched (D=R) NOD-NOR.

*Legend: green (HR <1.0), yellow (HR 1-1.2), orange (HR 1.2-1.4), red (HR >1.4)*

| Hazard ratio for all-cause graft loss (95% CI) | | | | |
| --- | --- | --- | --- | --- |
| DR Weight Mismatch (kg) | NOD-NOR | OD-NOR | NOD-OR | OD-OR |
| >30 (D>R) | 0.98 (0.93-1.04) | 1.00 (0.97-1.04) | 0.77 (0.41-1.42) | 1.07 (0.98-1.17) |
| 10-30 (D>R) | 0.99 (0.96-1.02) | 1.01 (0.97-1.05) | 1.03 (0.91-1.16) | 1.05 (0.99-1.12) |
| < 10 (D=R) | Ref. | 1.02 (0.97-1.08) | 0.99 (0.94-1.03) | 1.05 (1.00-1.11) |
| 10-30 (D<R) | **1.03 (1.01-1.07)** | 1.06 (0.92-1.21) | 1.02 (0.98-1.05) | **1.16 (1.10-1.24)** |
| >30 (D<R) | **1.19 (1.14-1.25)** | **1.73 (1.08-2.79)** | **1.14 (1.11-1.18)** | **1.27 (1.15-1.40)** |

Abbreviations: NOD-NOR, non-obese donor-non-obese recipient; OD-NOR, obese-donor-non-obese recipient; NOD-OR, non-obese donor-obese recipient; OD-OR, obese-donor-obese-recipient

We adjusted for known literature predictors of graft loss, including donor and recipient age, race, sex, recipient end-stage kidney disease (ESKD) cause, cold ischemia time (CIT), dialysis vintage, pre-emptive status, previous kidney transplant, human leukocyte antigen (HLA) mismatch, peak panel reactive antibody (PRA), and recipient medical comorbidities (coronary artery disease, hypertension, peripheral vascular disease, type 2 diabetes)*.*

**Supplemental Table 3:** Odds ratios for delayed graft function for each DR weight mismatch category stratified by DR obesity status. Reference category used for all DR obesity pairings was weight-matched (D=R) NOD-NOR.

*Legend: green (HR <1.0), yellow (HR 1-1.2), orange (HR 1.2-1.4), red (HR >1.4)*

| Odds ratio for DGF (95% CI) | | | | |
| --- | --- | --- | --- | --- |
| DR Weight Mismatch (kg) | NOD-NOR | OD-NOR | NOD-OR | OD-OR |
| >30 (D>R) | 0.95 (0.86-1.06) | **1.46 (1.37-1.55)** | 1.86 (0.74-4.70) | **2.33 (2.06-2.64)** |
| 10-30 (D>R) | 1.00 (0.95-1.06) | **1.29 (1.21-1.39)** | **1.33 (1.09-1.62)** | **1.98 (1.80-2.18)** |
| < 10 (D=R) | Ref. | **1.37 (1.25-1.51)** | **1.53 (1.42-1.66)** | **1.90 (1.75-2.06)** |
| 10-30 (D<R) | 1.01 (0.96-1.07) | **1.34 (1.04-1.72)** | **1.40 (1.32-1.49)** | **1.93 (1.75-2.14)** |
| >30 (D<R) | **1.24 (1.14-1.34)** | 1.96 (0.81-4.75) | **1.62 (1.53-1.71)** | **2.45 (2.08-2.88)** |

Abbreviations: NOD-NOR, non-obese donor-non-obese recipient; OD-NOR, obese-donor-non-obese recipient; NOD-OR, non-obese donor-obese recipient; OD-OR, obese-donor-obese-recipient; DGF, delayed graft function.

We adjusted for known literature predictors of graft loss, including donor and recipient age, race, sex, recipient end-stage kidney disease (ESKD) cause, cold ischemia time (CIT), dialysis vintage, pre-emptive status, previous kidney transplant, human leukocyte antigen (HLA) mismatch, peak panel reactive antibody (PRA), and recipient medical comorbidities (coronary artery disease, hypertension, peripheral vascular disease, type 2 diabetes)*.*

**Supplemental Table 4:** Odds ratios for early graft loss for each DR weight mismatch category stratified by DR obesity status. Reference category used for all DR obesity pairings was weight-matched (D=R) NOD-NOR.

*Legend: green (HR <1.0), yellow (HR 1-1.2), orange (HR 1.2-1.4), red (HR >1.4)*

| Hazard ratio for Early graft loss (95% CI) | | | | |
| --- | --- | --- | --- | --- |
| DR Weight Mismatch (kg) | NOD-NOR | OD-NOR | NOD-OR | OD-OR |
| >30 (D>R) | 0.98 (0.76-1.28) | **1.19 (1.01-1.40)** | 1.76 (0.24-13.14) | **1.62 (1.18-2.23)** |
| 10-30 (D>R) | 0.90 (0.77-1.05) | **1.21 (1.01-1.44)** | 0.86 (0.48-1.54) | 1.19 (0.91-1.56) |
| < 10 (D=R) | Ref. | 1.24 (0.98-1.58) | 1.09 (0.88-1.35) | 1.13 (0.90-1.42) |
| 10-30 (D<R) | 1.00 (0.86-1.17) | 1.12 (0.57-2.18) | 1.14 (0.97-1.34) | **1.54 (1.19-1.98)** |
| >30 (D<R) | **1.50 (1.22-1.84)** | **7.30 (2.19-24.30)** | **1.32 (1.14-1.53)** | **1.79 (1.21-2.67)** |

Abbreviations: NOD-NOR, non-obese donor-non-obese recipient; OD-NOR, obese-donor-non-obese recipient; NOD-OR, non-obese donor-obese recipient; OD-OR, obese-donor-obese-recipient

We adjusted for known literature predictors of graft loss, including donor and recipient age, race, sex, recipient end-stage kidney disease (ESKD) cause, cold ischemia time (CIT), dialysis vintage, pre-emptive status, previous kidney transplant, human leukocyte antigen (HLA) mismatch, peak panel reactive antibody (PRA), and recipient medical comorbidities (coronary artery disease, hypertension, peripheral vascular disease, type 2 diabetes)*.*

**Supplemental Table 5**: Hazard ratios for DCGL by DR obesity status excluding donors and recipients with BMI <18

|  | DCGL  Hazard Ratio  (95% CI) | All-cause graft loss  Hazard Ratio  (95% CI) | DGF  Odds Ratio  (95% CI) | Early (≤30 day) graft loss  Odds Ratio  (95% CI) |  |
| --- | --- | --- | --- | --- | --- |
| NOD-NOR | Ref. | Ref. | Ref. | Ref. | |
| OD-NOR | 1.02 (0.98-1.06) | 1.00 (0.98-1.02) | **1.36 (1.31-1.42)** | **1.19 (1.07-1.33)** | |
| NOD-OR | **1.15 (1.11-1.19)** | **1.05 (1.02-1.07)** | **1.49 (1.44-1.55)** | **1.19 (1.07-1.32)** | |
| OD-OR | **1.25 (1.20-1.31)** | **1.09 (1.05-1.12)** | **2.00 (1.90-2.10)** | **1.34 (1.18-1.53)** | |

Abbreviations: NOD-NOR, non-obese donor-non-obese recipient; OD-NOR, obese-donor-non-obese recipient; NOD-OR, non-obese donor-obese recipient; OD-OR, obese-donor-obese-recipient; DCGL, death-censored graft loss; DGF, delayed graft function.

We adjusted for known literature predictors of graft loss, including donor and recipient age, race, sex, recipient end-stage kidney disease (ESKD) cause, cold ischemia time (CIT), dialysis vintage, pre-emptive status, previous kidney transplant, human leukocyte antigen (HLA) mismatch, peak panel reactive antibody (PRA), and recipient medical comorbidities (coronary artery disease, hypertension, peripheral vascular disease, type 2 diabetes)*.*

**Supplemental Table 6:** Hazard ratios for death-censored graft loss (DCGL) for each DR weight mismatch category stratified by DR obesity status. Reference category used for each DR obesity pairing was weight-matched (D=R) DR from the same group.

|  | Hazard ratio for DCGL (95% CI) | | | | |
| --- | --- | --- | --- | --- | --- |
| DR Weight Mismatch  (kg) | All DR  N = 238,895 | NOD-NOR  N = 123,449 | OD-NOR  N = 38,969 | NOD-OR  N = 53,964 | OD-OR  N = 22,513 |
| >30 (D>R) | **0.95 (0.91-0.99)** | **0.83 (0.76-0.91)** | **0.89 (0.82-0.98)** | 0.50 (0.19-1.34) | 1.04 (0.90-1.19) |
| 10-30 (D>R) | **0.96 (0.93-0.99)** | **0.94 (0.89-0.98)** | 0.93 (0.85-1.02) | 1.08 (0.90-1.29) | 0.96 (0.87-1.07) |
| < 10 (D=R) | Ref. | Ref. | Ref. | Ref. | Ref. |
| 10-30 (D<R) | **1.10 (1.06-1.13)** | **1.13 (1.08-1.18)** | 1.03 (0.84-1.27) | 1.07 (0.99-1.15) | 1.11 (1.00-1.23) |
| >30 (D<R) | **1.28 (1.24-1.33)** | **1.42 (1.32-1.52)** | 1.78 (0.92-3.45) | **1.26 (1.16-1.37)** | **1.21 (1.04-1.41)** |

Abbreviations: NOD-NOR, non-obese donor-non-obese recipient; OD-NOR, obese-donor-non-obese recipient; NOD-OR, non-obese donor-obese recipient; OD-OR, obese-donor-obese-recipient; DCGL, death-censored graft loss.

We adjusted for known literature predictors of graft loss, including donor and recipient age, race, sex, recipient end-stage kidney disease (ESKD) cause, cold ischemia time (CIT), dialysis vintage, pre-emptive status, previous kidney transplant, human leukocyte antigen (HLA) mismatch, peak panel reactive antibody (PRA), and recipient medical comorbidities (coronary artery disease, hypertension, peripheral vascular disease, type 2 diabetes)*.*

**Supplemental Table 7:** Hazard ratios for death-censored graft loss (DCGL) for each DR height mismatch category stratified by DR obesity status

|  | Hazard ratio for DCGL (95% CI) | | | | |
| --- | --- | --- | --- | --- | --- |
| DR Height Mismatch  (cm) | All DR | NOD-NOR | OD-NOR | NOD-OR | OD-OR |
| >15 (D>R) | **0.91 (0.87-0.94)** | **0.89 (0.84-0.95)** | 0.90 (0.81-1.01) | 0.95 (0.87-1.04) | 0.93 (0.81-1.07) |
| 5-15 (D>R) | 0.96 (0.93-1.00) | 0.97 (0.92-1.02) | 0.99 (0.90-1.09) | 0.96 (0.89-1.04) | 1.02 (0.91-1.15) |
| < 5 (D=R) | Ref. | Ref. | Ref. | Ref. | Ref. |
| 5-15 (D<R) | **1.06 (1.02-1.09)** | **1.08 (1.03-1.15)** | 1.01 (0.92-1.11) | 1.07 (0.99-1.15) | 1.08 (0.96-1.21) |
| >15 (D<R) | **1.24 (1.19-1.28)** | **1.25 (1.18-1.33)** | **1.20 (1.08-1.33)** | **1.27 (1.16-1.38)** | **1.20 (1.05-1.36)** |

Abbreviations: NOD-NOR, non-obese donor-non-obese recipient; OD-NOR, obese-donor-non-obese recipient; NOD-OR, non-obese donor-obese recipient; OD-OR, obese-donor-obese-recipient; DCGL, death-censored graft loss.

We adjusted for known literature predictors of graft loss, including donor and recipient age, race, sex, recipient end-stage kidney disease (ESKD) cause, cold ischemia time (CIT), dialysis vintage, pre-emptive status, previous kidney transplant, human leukocyte antigen (HLA) mismatch, peak panel reactive antibody (PRA), and recipient medical comorbidities (coronary artery disease, hypertension, peripheral vascular disease, type 2 diabetes)*.*

**Supplemental Table 8:** Hazard ratios for death-censored graft loss (DCGL) for each DR weight mismatch category stratified by DR obesity status for **deceased** donors

|  | Hazard ratio for DCGL (95% CI)  (DECEASED) | | | | |
| --- | --- | --- | --- | --- | --- |
| DR Weight Mismatch  (kg) | All DR | NOD-NOR | OD-NOR | NOD-OR | OD-OR |
| >30 (D>R) | 0.96 (0.92-1.01) | **0.82 (0.75-0.90)** | 0.91 (0.82-1.02) | 0.61 (0.20-1.90) | 1.05 (0.91-1.22) |
| 10-30 (D>R) | 0.97 (0.93-1.01) | 0.95 (0.90-1.0) | 0.93 (0.82-1.02) | 1.08 (0.89-1.32) | 0.96 (0.85-1.09) |
| < 10 (D=R) | Ref. | Ref. | Ref. | Ref. | Ref. |
| 10-30 (D<R) | **1.11 (1.07-1.15)** | **1.15 (1.09-1.21)** | 1.08 (0.83-1.40) | 1.07 (0.98-1.17) | **1.15 (1.02-1.31)** |
| >30 (D<R) | **1.33 (1.27-1.38)** | **1.51 (1.40-1.62)** | **2.07 (1.02-4.19)** | **1.25 (1.14-1.37)** | **1.39 (1.16-1.66)** |

Abbreviations: NOD-NOR, non-obese donor-non-obese recipient; OD-NOR, obese-donor-non-obese recipient; NOD-OR, non-obese donor-obese recipient; OD-OR, obese-donor-obese-recipient; DCGL, death-censored graft loss.

We adjusted for known literature predictors of graft loss, including donor and recipient age, race, sex, recipient end-stage kidney disease (ESKD) cause, cold ischemia time (CIT), dialysis vintage, pre-emptive status, previous kidney transplant, human leukocyte antigen (HLA) mismatch, peak panel reactive antibody (PRA), and recipient medical comorbidities (coronary artery disease, hypertension, peripheral vascular disease, type 2 diabetes)*.*

**Supplemental Table 9:** Hazard ratios for death-censored graft loss (DCGL) for each DR weight mismatch category stratified by DR obesity status for **living** donors

|  | Hazard ratio for DCGL (95% CI)  (LIVING) | | | | |
| --- | --- | --- | --- | --- | --- |
| DR Weight Mismatch  (kg) | All DR | NOD-NOR | OD-NOR | NOD-OR | OD-OR |
| >30 (D>R) | **0.87 (0.80-0.95)** | 0.87 (0.73-1.03) | **0.80 (0.65-0.98)** | 0.28 (0.39-2.00) | 0.97 (0.64-1.47) |
| 10-30 (D>R) | **0.91 (0.86-0.97)** | **0.89 (0.81-0.97)** | 0.92 (0.78-1.09) | 1.07 (0.68-1.68) | 0.97 (0.77-1.22) |
| < 10 (D=R) | Ref. | Ref. | Ref. | Ref. | Ref. |
| 10-30 (D<R) | **1.11 (1.05-1.17)** | **1.12 (1.02-1.22)** | 0.99 (0.69-1.42) | 1.06 (0.89-1.26) | 1.09 (0.90-1.33) |
| >30 (D<R) | **1.26 (1.18-1.35)** | **1.29 (1.09-1.53)** | 0.94 (0.13-6.75) | **1.32 (1.10-1.59)** | 1.02 (0.76-1.35) |

Abbreviations: NOD-NOR, non-obese donor-non-obese recipient; OD-NOR, obese-donor-non-obese recipient; NOD-OR, non-obese donor-obese recipient; OD-OR, obese-donor-obese-recipient; DCGL, death-censored graft loss.

We adjusted for known literature predictors of graft loss, including donor and recipient age, race, sex, recipient end-stage kidney disease (ESKD) cause, cold ischemia time (CIT), dialysis vintage, pre-emptive status, previous kidney transplant, human leukocyte antigen (HLA) mismatch, peak panel reactive antibody (PRA), and recipient medical comorbidities (coronary artery disease, hypertension, peripheral vascular disease, type 2 diabetes)*.*
